# Emerging Therapeutic Strategies for Targeting Hyaluronan in Metabolic Disease from Molecular Design to Clinical Translation

**DOI:** 10.3390/ijms27219712

**Published:** 2026-10-29

**Authors:** Mingpeng Feng, Xin Hu, Hong Zhou

**Affiliations:** 1 Medicine & Technology College, Zunyi Medical University, Zunyi 563000, China; destinyx2023@outlook.com; 2 Department of Cancer Biology, The University of Texas M.D. Anderson Cancer Center, Houston, TX 77030, USA; 3 USDA/ARS Children’s Nutrition Research Center, Department of Pediatrics, Baylor College of Medicine, Houston, TX 77030, USA

**Keywords:** hyaluronan, therapeutic targeting, drug delivery, metabolic disease, patient stratification, translational barriers

## Abstract

Hyaluronan (HA) is increasingly implicated as a contributor to metabolic disease; however, the strongest causal evidence still derives from preclinical models, and no HA-directed treatment has reached the metabolic clinic. That gap points to a weakness in the underlying therapeutic logic. Clinical results for systemic removal of HA have been mixed rather than uniformly negative. In the phase II HALO-202 trial, PEGPH20 improved progression-free survival in patients with hyaluronan-high pancreatic tumors, whereas the phase III HALO-301 trial, which enrolled only hyaluronan-high patients, showed no overall survival benefit; depletion alone was therefore insufficient, and this outcome cannot be attributed to unselected treatment or to molecular size. Its biological activity is not determined by molecular size alone. Concentration, preparation purity, receptor context, tissue location, and the balance between high-molecular-weight HA (HMW-HA, generally above 1000 kDa) and smaller fragments, which range from oligosaccharides of about ten saccharides or fewer to low-molecular-weight HA in between, jointly influence whether the matrix mainly protects or injures metabolic tissues. The argument here is that the field should move away from bulk depletion and toward precision delivery and biomarker-guided patient selection. The therapeutic landscape is organized around four strategies: pharmacological inhibition of HA synthesis, enzymatic degradation, HA-based biomaterials, and defined-molecular-weight HA fragments. Throughout, we distinguish interventions that modify endogenous HA biology from platforms that use HA as a carrier or scaffold, because improved drug delivery or wound healing does not by itself demonstrate correction of pathological HA signaling. Their translational obstacles are then assessed, including patient stratification by HA size and CD44 signature, standardization of size-resolved HA quantification, thromboembolic risk, manufacturing and regulatory hurdles for drug–device combinations, and the design of trials with composite metabolic endpoints. HA is best viewed as an amplifier of metabolic stress rather than a bystander, and the route to clinical translation lies in restoring the equilibrium between HA synthesis and degradation rather than eliminating the molecule, a goal that remains a translational hypothesis rather than a demonstrated clinical outcome.

## 1. Introduction

Two of the most prevalent chronic metabolic diseases, obesity and type 2 diabetes (T2D), carry much of their burden through low-grade inflammation that remodels the extracellular matrix (ECM) in metabolically active tissues [1,2]. The ECM is not an inert scaffold but a dynamic structure whose composition and mechanical properties govern nutrient sensing, immune-cell recruitment, and the transduction of insulin signals [1,3]. Hyaluronan (HA) stands out among matrix macromolecules because its synthesis responds to nutrient supply, its fragmentation tracks tissue stress, and its receptors are present on nearly every metabolic and immune cell [4,5]. These observations fit a single framework in which HA amplifies metabolic stress without initiating it. Nutrient excess and tissue injury raise HA levels, and the resulting matrix remodeling spreads inflammation and insulin resistance, positioning the HA axis as both a biomarker and a node for intervention [6,7]. The World Health Organization estimates that more than 650 million adults are living with obesity and over half a billion have diabetes, which points to an urgent need for therapies directed at pathological matrix remodeling. The review examines HA metabolism and receptor signaling, organ-specific pathophysiology, biomarker value, and treatment options, and it closes with a forward-looking view of the main obstacles to clinical implementation.

Among the glycosaminoglycans, HA is an unsulfated linear polymer assembled from repeating D-glucuronic acid and N-acetyl-D-glucosamine units and produced at the cell surface through the action of three hyaluronan synthases (HAS1-3) [8,9,10,11,12]. In healthy tissue, HA exists largely as high-molecular-weight HA (HMW-HA), which sustains hydration, keeps the tissue compliant, and holds the microenvironment in an immunosuppressive state [4,8]. Under metabolic stress, HA synthesis rises while hyaluronidases and reactive oxygen species cleave the polymer into low-molecular-weight HA (LMW-HA) and oligosaccharides. In specific experimental and pathological contexts, depending on fragment size, local concentration, and preparation purity, these fragments can behave as damage-associated molecular patterns that activate CD44 and Toll-like receptors (TLRs) to promote inflammatory signaling cascades [8,13,14,15,16,17].

HA has served clinical medicine for decades as a viscosupplement, a dermal filler, and a biomaterial [18,19]. Its contribution to metabolic disease, however, has become clear only recently, through genetic and pharmacological approaches [20,21,22]. The strength of that evidence differs by context. Causality is best supported in preclinical models, in which genetic deletion or pharmacological inhibition of HA synthesis or of CD44 changes insulin sensitivity, β-cell survival, and tissue inflammation [20,21,22,23,24,25]. In humans the evidence remains largely associative, because circulating HA and endothelial glycocalyx markers track obesity, insulin resistance, and renal or vascular complications without establishing the direction of the effect or its mechanism [26,27,28]. We therefore use causal language only where genetic or pharmacological manipulation supports it and describe the remaining findings as associations. In the review we bring together what is now known about HA metabolism and receptor signaling, and the HA-directed therapeutic strategies now emerging, from inhibiting HA synthesis to building HA-based delivery systems. We end by setting out the uncertainties that must be resolved for HA-based interventions to reach metabolic medicine. A comprehensive literature search was conducted in PubMed (search strategy detailed in Supplementary Table S1), from which records were screened and selected for inclusion (Supplementary Table S2). The sharp increase in retrieved publications over recent decades underscores the accelerating interest in this field (Supplementary Figure S1).

## 2. Hyaluronan Biology: Synthesis, Degradation, and Receptor Signaling

### 2.1. Biosynthesis and Catabolism

Three plasma-membrane enzymes, HAS1, HAS2, and HAS3, polymerize HA from the nucleotide-sugar donors UDP-glucuronic acid and UDP-N-acetylglucosamine. Because UDP-glucose dehydrogenase and glutamine:fructose-6-phosphate aminotransferase lie upstream, HA output is coupled to glucose and glutamine metabolism [8,12]. HAS2 is the most dynamically regulated isoform and is controlled after translation by O-GlcNAcylation, which links hexosamine flux directly to HA synthesis [12]. The three isoforms are not interchangeable. They differ in enzymatic properties and in the polymer sizes they generate, and their expression is governed at the transcriptional, post-transcriptional, and post-translational levels, with a natural antisense transcript regulating HAS2 [29,30]. Inflammatory cytokines and growth factors induce HAS activity, PPAR-γ ligands repress it in certain cell types, and oxidized lipoproteins modulate it [31,32,33]. Energy status also influences HA synthesis. AMP-activated protein kinase (AMPK) suppresses HAS2 activity and HA generation in human aortic smooth muscle cells, tying energy sensing directly to matrix synthesis [34]. The isoforms also produce different-sized polymers. HAS2 tends to generate large HA, HAS3 generates shorter chains, and HAS1 has been linked to HMW-HA and tissue-protective responses [35,36]. Consistently with this context-dependent behavior, enforced HAS1 expression dampens inflammation in experimental wounds, whereas LMW-HA generated by HAS3 is associated with worsened acute lung injury in preclinical ventilated rat models [35,36].

HA breakdown is equally dynamic. Hyaluronidases, including HYAL1, HYAL2, and the secreted PH20 among them, cut the polymer apart, while cell-surface receptors take up the resulting fragments for intracellular degradation [8,9,14,37]. The human genome contains six hyaluronidase-like genes, and the encoded enzymes differ in tissue distribution, subcellular location, substrate preference, and the fragments they produce [37,38]. HYAL1 is the principal lysosomal hyaluronidase and degrades oligosaccharides to tetrasaccharides, whereas HYAL2 is anchored at the cell surface, associates with CD44, and initiates cleavage of HMW-HA in the extracellular space [37,38]. HYAL3 shows little or no catalytic activity toward HA and may instead regulate the other enzymes, whereas HYAL4 acts on chondroitin sulfate and is not a hyaluronidase in the strict sense [37,38]. PH20 (SPAM1) is expressed in the testis and provides the enzyme used therapeutically as PEGPH20 [38,39]. Two further players, the cell-surface hyaluronidase TMEM2 (CEMIP2) and the secreted protein CEMIP (KIAA1199), depolymerize extracellular HA and expand the range of fragment sizes that cells encounter [40,41]. Reactive oxygen species and transition-metal-driven oxidation also fragment HA without enzymatic activity—a route particularly relevant in inflamed, metabolically stressed tissues as well as in radiation-injured skin [14,42,43]. The kinetics of HA turnover in tissues and circulation have been mapped in detail, with intravenously injected HA being cleared rapidly from the bloodstream [44,45]. Furthermore, differentiated macrophages synthesize and secrete a complex ECM, showing that infiltrating leukocytes both shape and respond to HA remodeling in metabolic tissues [46]. Supplementary Table S3 summarizes these enzymatic and non-enzymatic degradation routes.

### 2.2. Molecular-Weight-Dependent Signaling

Molecular size strongly influences the biological behavior of HA, but its effects depend on tissue, inflammatory state, concentration, purity, and receptor context. Fragments are now regarded as an information-rich signaling system whose output is shaped by these variables [8,9,47,48,49,50]. Measurements in biological fluids show that HA content and its size distribution vary among tissues, providing a frame of reference for interpreting size-dependent signaling [51]. In most settings, HMW-HA is anti-inflammatory and immunosuppressive. It calms allergic airway inflammation, improves hemodynamics in preclinical abdominal sepsis, reduces M1 macrophage polarization and IL-1β-driven synovial inflammation, and dampens neuroinflammatory responses after nerve injury [52,53,54,55]. Conversely, LMW-HA and HA oligosaccharides have frequently been linked to pro-inflammatory signaling, although whether they elicit an inflammatory response depends critically on fragment concentration, preparation purity, endotoxin status, and cellular context [16,17,56]. Where pro-inflammatory signaling occurs, extracellular superoxide dismutase 3 can reduce HA-fragment-mediated skin inflammation, marking oxidative fragmentation as a context-dependent regulatory checkpoint [43]. Cross-linking of HA chains by inter-alpha-inhibitor heavy chains may be protective, stabilizing the HA matrix and keeping pro-inflammatory fragments out of reach [57].

Operational definitions are needed because the same labels are applied to different size ranges in the literature, and this inconsistency is one reason why reports of HMW-HA and LMW-HA activity appear to conflict [16]. In this review, HMW-HA denotes polymers above about 1000 kDa, which corresponds to the size of the native polymer; HA oligosaccharides denote fragments of about ten saccharides or fewer, the size below which monovalent rather than multivalent CD44 engagement predominates; and LMW-HA denotes everything between these limits, a range that in practice spans roughly 10–1000 kDa and is often divided further into small fragments of about 10–100 kDa and intermediate polymers of 100–1000 kDa [16,48]. These boundaries are conventions rather than biological thresholds, and the reported activity of a preparation depends on its size distribution, its concentration, bound proteins, receptor density, and the tissue in which it is tested [16,56,58], as is shown in Table 1.

Preparation purity is an equally important variable that is not always reported. Pharmaceutical-grade HA and HA fragments free of endotoxin did not stimulate TNF-α, interleukin-12, or co-stimulatory molecules in macrophages and dendritic cells, whereas preparations contaminated with endotoxin did so even after polymyxin B treatment [17]. Reported pro-inflammatory effects of HA fragments should therefore be interpreted together with the purity and endotoxin content of the preparation, and studies that omit these parameters deserve caution.

### 2.3. Receptors: CD44, RHAMM, and Toll-like Receptors

CD44 is the dominant HA receptor carried by leukocytes and stromal cells, and it sits at the center of HA-driven inflammation and its resolution, where CD44-hyaluronan interactions guide leukocyte trafficking in acute inflammation [59,60,61]. Early studies identified CD44 as the principal surface receptor that binds hyaluronate, and its roles have since grown beyond cell adhesion into signal transduction [62,63]. CD44-dependent adhesion to HA matrices controls T-lymphocyte migration, and inflammatory cytokines, tumor necrosis factor-alpha (TNF-α) among them, induce the ability of monocytes to bind HA [64,65]. Binding HA to CD44 is tightly controlled. A labile disulfide bond in the binding groove makes it redox-sensitive, chondroitin sulfate plus the sialidase Neu1 influence it, and inflammatory cytokines, especially IL-1β, modulate it [66,67,68,69]. What CD44 engagement ultimately does depends on context. In models where HA fragments trigger inflammation, this signaling requires CD44, which also drives T-helper type 1 differentiation via an Annexin A1/Akt/mTOR pathway [15,70]. Conversely, CD44 deficiency worsens LPS-triggered pulmonary inflammation and lung injury evoked by hyperoxia, and giving HA beforehand suppresses TLR4 signaling through CD44 [60,71,72,73,74]. The receptor can be exploited therapeutically too, since it strengthens induction of regulatory T cells in allergen-specific immunotherapy [75]. A second HA receptor, the receptor for hyaluronan-mediated motility (RHAMM/HMMR), connects to obesity-related tissue injury [76], while TLR2 and TLR4 have been reported in several experimental systems to participate in sensing small fragments of HA, an interaction whose output remains sensitive to preparation purity and biological context [13,15,77] (Table 2).

### 2.4. Hyaluronan-Binding Proteins: Regulators of HA-Receptor Interactions

HA does not work through its receptors alone. A set of hyaluronan-binding proteins also organizes the pericellular matrix and modulates receptor engagement [81]. TSG-6 moves inter-alpha-inhibitor heavy chains onto HA, an enzymatic transfer that stabilizes HA-rich matrices and keeps pro-inflammatory fragments from being easily accessed. When this protective mechanism goes awry, metabolic tissue injury may result [57]. Versican and aggrecan, two further HA-binding proteoglycans, cross-link HA networks and affect leukocyte adhesion and the compliance of tissues [81].

### 2.5. Hyaluronan Synthesis as a Metabolic Node

**Figure 1:**
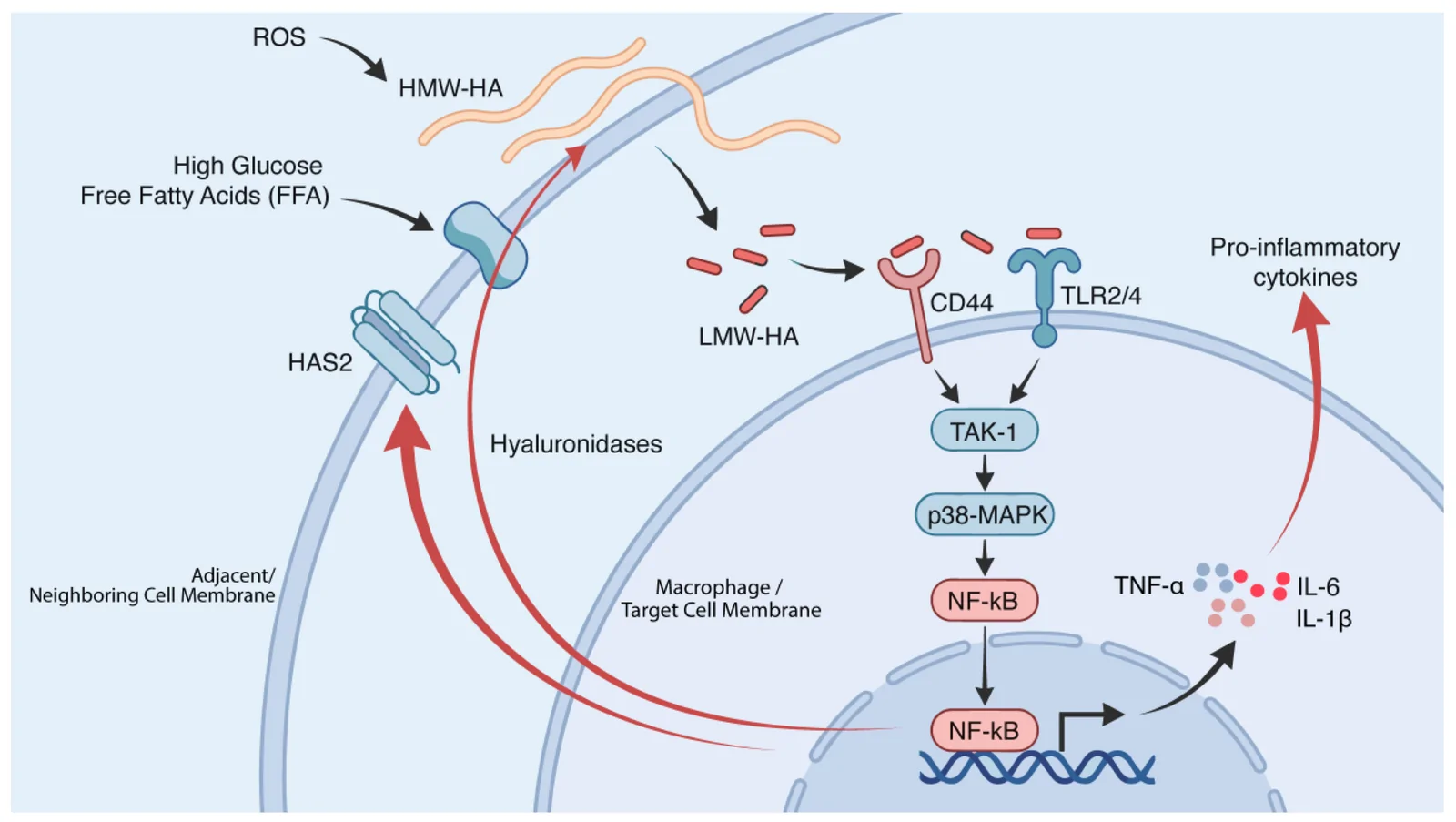
Biosynthesis, enzymatic and oxidative degradation, and signaling pathways of hyaluronan in metabolic regulation. Hyaluronan (HA) biosynthesis is coupled to intracellular nutrient flux. Under metabolic stress, elevated levels of glucose and free fatty acids (FFAs) upregulate membrane-bound HAS2 to synthesize high-molecular-weight hyaluronan (HMW-HA). In the extracellular space, non-enzymatic cleavage by reactive oxygen species (ROS) alongside depolymerization by hyaluronidases breaks down HMW-HA into low-molecular-weight fragments (LMW-HA). Depending on local concentration, preparation purity, and receptor context, these LMW-HA fragments have been reported to act as pro-inflammatory ligands that engage CD44 and Toll-like receptors 2 and 4 (TLR2/4), sequentially activating TAK-1 and p38-MAPK cascades to promote the nuclear translocation of NF-κB. Once inside the nucleus, NF-κB can drive the transcription and release of pro-inflammatory cytokines (TNF-α, IL-6, and IL-1β). Concurrently, activated NF-κB is proposed to form a positive feed-forward loop (red arrows) that transcriptionally upregulates HAS2 and hyaluronidases, potentially sustaining pathological HA fragmentation, chronic matrix remodeling, and metabolic inflammation. Size classes in this figure are operational: HMW-HA generally exceeds 1000 kDa, HA oligosaccharides comprise roughly ten saccharides or fewer, and LMW-HA covers the range between. Fragment-induced inflammatory responses require careful interpretation because concentration, preparation purity, and endotoxin contamination can determine whether a response is observed [16,17].

Beyond consuming matrix precursors, HA synthesis feeds directly into core carbon metabolism. UDP-glucuronic acid is generated from glucose-6-phosphate, and UDP-N-acetylglucosamine is produced through the hexosamine pathway, which means HA production draws directly on glycolytic intermediates and glutamine-derived amino groups [8,12]. Because HA synthesis depends heavily on these nucleotide-sugar donors, changes in HA metabolism perturb cellular bioenergetics. In brown adipose tissue, inhibiting HA synthesis with 4-MU diverts glucose flux toward glycolysis and supports mitochondrial respiration [21], whereas remodeling of the ECM can govern glucose uptake by destabilizing thioredoxin-interacting protein (TXNIP) [3]. Treatment with hyaluronidase also induces metabolic shifts during acute inflammation [82]. The hexosamine route that supplies UDP-N-acetylglucosamine for HA synthesis has long been linked to insulin resistance and diabetic complications, which underscores the metabolic significance of HA precursor flux [83]. Taken together, these observations indicate that whether HA synthesis outpaces its degradation shapes both the extracellular HA pool and intracellular glucose handling, a connection that now links matrix biology with metabolic regulation [3,9,21] (Figure 1). When insulin resistance or hyperglycemia is present, excess HA synthesis may place further load on the hexosamine pathway and increase intracellular metabolic pressure. This coupling appears most pronounced in tissues with high matrix turnover, adipose tissue and liver among them, although its quantitative contribution to metabolic dysfunction has not been established.

### 2.6. Molecular-Weight-Dependent Actions of HA: HMW-HA Versus LMW-HA

CD44 carries a conformationally regulated HA-binding site, and biophysical work in artificial membrane systems shows that low- and high-molecular-weight fragments engage CD44 with binding properties that differ, stemming from differences in avidity and receptor clustering [84,85]. Force measurements further indicate that the mechanical interaction between HA and its receptor scales with molecular weight. These observations define a graded rather than a binary response, because the number of CD44-binding sites engaged by a single chain, and therefore the signaling outcome, depends on chain length, receptor density, and the presence of HA-binding proteins such as TSG-6 and inter-alpha-inhibitor [57,81]. The relative contribution of size, concentration, and receptor context to metabolic outcomes in vivo has not been quantified, and the size-based statements in this review should be read against that limitation.

## 3. Therapeutic Targeting of Hyaluronan in Metabolic Disease

Strategies that involve hyaluronan are not equivalent, and the distinction runs through the rest of this review. One group modifies endogenous HA biology: inhibition of HA synthesis, enzymatic degradation of the matrix, and supplementation with defined-size fragments or HMW-HA all change the amount, the size, or the signaling behavior of endogenous HA. A second group uses HA as a material. Hydrogels, microneedles, and HA-coated nanoparticles exploit the biocompatibility, CD44 affinity, and enzyme-responsive chemistry of the polymer to deliver another agent, and in these systems the therapeutic effect may come from the payload, from the carrier, or from the combination. Improved insulin delivery or faster wound closure therefore does not by itself demonstrate that pathological HA signaling has been corrected. Attributing an effect to HA biology requires an HA-relevant readout, such as HA content, fragment size, or receptor engagement, together with controls that separate the carrier from the payload. The tables below indicate the category to which each strategy belongs. Therapeutic strategies and their translational characteristics are organized in Table 3, and representative preclinical and clinical studies detailing specific disease models, dosing regimens, primary outcomes, and study limitations are compiled in Supplementary Table S4.

### 3.1. Pharmacological Inhibition of Hyaluronan Synthesis

The orally bioavailable drug 4-MU (hymecromone) is already approved clinically for biliary spasm. Its best-characterized mechanism is substrate depletion: 4-MU is conjugated to 4-methylumbelliferyl glucuronide (4-MUG), which consumes UDP-glucuronic acid and thereby limits the precursor pool available to all three HAS isoforms. This mechanism is not isoform-selective, and 4-MUG itself also inhibits HA synthesis, which explains why the drug is active despite rapid glucuronidation [86]. Reduced HAS2 expression has been reported in several 4-MU-treated models, including pancreatic islets, brown adipose tissue, and kidney, but the effect is tissue- and context-dependent and should not be assumed to occur uniformly or to explain all of the reported metabolic benefits. Across metabolic models, 4-MU preserves glycemic control and β-cell survival in obesity-linked T2D and autoimmune insulitis [20,22], raises thermogenesis in BAT and insulin sensitivity [21,87], slows diabetic kidney disease [88], guards the kidney against ischemia–reperfusion injury [89], and dampens acute inflammation in lung and brain [90,91]. Transcriptomic and public-data analyses point to additional targets beyond HA, which complicates mechanistic attribution but widens the drug’s therapeutic reach [92]. Isoform-selective inhibitors would allow more selective suppression of pathological HA synthesis, but no HAS2- or HAS3-selective compound has been validated in humans, and 4-MU remains the only inhibitor with clinical experience [88,94]. Because 4-MU also affects UDP-glucuronosyltransferases and the pool of glycosaminoglycan precursors, part of its metabolic effect may be independent of HA synthesis, which complicates attribution in single-agent studies [92]. Importantly, increased HA production is not uniformly detrimental. Adipose-specific *Has2* overexpression improved systemic glucose homeostasis in a mouse study [130], indicating that the consequences of HA accumulation depend on its tissue source and biological context. These findings caution against extrapolating the benefits of systemic 4-MU treatment to a general requirement for suppressing HA synthesis in all metabolic tissues.

### 3.2. Enzymatic Degradation of Hyaluronan

PEGPH20, a pegylated hyaluronidase that depletes HA, offers an alternative. In DIO mice it reduces fat mass and restores muscle insulin sensitivity [23,25]. When highly purified, recombinant PH20 does not provoke inflammation [39], and in sepsis models, hyaluronidase therapy changes acute inflammation and metabolism [82]. Removing the parent polymer is not equivalent to removing HA-derived biological activity. Enzymatic cleavage generates fragments whose size determines whether they are cleared or whether they engage CD44, TLR2/4, or RHAMM; small fragments are taken up and degraded in the lysosome, whereas larger fragments can persist in the matrix and retain signaling activity [9,14,37,38,40,41,131]. The therapeutic rationale for enzymatic degradation therefore rests on the balance between removing pathological matrix and generating fragments that may themselves be bioactive, together with the clearance capacity of the tissue. This is one reason why responses to PEGPH20 differ between tissues and why size-resolved analysis of HA fragments are needed to interpret them. Breaking HA down may also serve fibrotic disease, since a micellar hyaluronidase formulation reduced pulmonary fibrosis in a preclinical setting [94]. Oncology provides a cautionary lesson. In the HALO-202 phase II trial, PEGPH20 met its primary endpoints: progression-free survival improved overall (hazard ratio: 0.73; *p* = 0.049) and in the subgroup with hyaluronan-high tumors (hazard ratio: 0.51; *p* = 0.048), and the objective response rate in that subgroup was higher (45% versus 31%) without a significant difference in overall survival (11.5 versus 8.5 months) [80]. However, the subsequent HALO-301 phase III trial showed no benefit in overall survival (11.2 versus 11.5 months; hazard ratio: 1.00; *p* = 0.97) or progression-free survival (7.1 versus 7.1 months), although the objective response rate was higher with PEGPH20 (47% versus 36%) in this hyaluronan-high population [79]. Selection for hyaluronan-high tumors was therefore applied and was not sufficient on its own, and these trials do not establish molecular size as the reason for the clinical failure. Gene therapy built on hyaluronidase could provide sustained enzymatic HA depletion, although it remains at an early stage. The HALO-301 failure highlights the central challenges for HA-directed therapy: the limits of stratification by HA level alone, careful management of clot-related risk, and the recognition that removing HA alone may not suffice unless the underlying tumor or metabolic microenvironment is also addressed.

### 3.3. Hyaluronan-Based Biomaterials and Delivery Systems

HA-based biomaterials have grown into a leading platform for metabolic-disease applications, thanks to HA’s biocompatibility, its affinity for CD44, and its enzyme-responsive chemistry [9,95,131,132]. HA’s central role in wound healing has long been recognized and has shaped how HA-based dressings are designed [96]. For diabetic wound care, HA hydrogels with self-healing, reactive-oxygen-scavenging, glucose-responsive, antimicrobial, or drug- and exosome-loaded properties accelerate healing through better modulation of inflammation, promotion of angiogenesis, and restoration of the wound microenvironment [97,98,99,100,101,102,103]. Related glucose-responsive designs have also been explored in chitosan/PVA composite nanohydrogels that combine glucose depletion with photothermal and NO/H_2_S-mediated therapy to promote diabetic wound healing [133]. In diabetic foot ulcers, a pooled review of trials on HA and its derivatives found better healing than with standard dressings [104], and ointments combining HA with collagenase, along with hydrogen-enriched HA dressings, have proved useful for chronic ulcers and diabetic ulcers [105,106].

HA microneedle arrays dissolve quickly in the interstitial fluid of skin and have been built for glucose-responsive insulin delivery, giving closed-loop glycemic regulation in preclinical models [108,109,110,111]. Boronate-modified HA conjugates add glucose sensitivity to insulin formulations [112], and glucose-responsive microneedle patches releasing glucagon help prevent hypoglycemia [113]. HA microneedle patches also promote diabetic wound healing by carrying antimicrobial and immunomodulatory agents into the skin [134,135]. In obesity, microneedles made of HA and loaded with liraglutide, oleanolic acid, semaglutide, hesperidin, or photothermal agents give sustained transdermal delivery and, in certain designs, trigger browning of white adipose tissue [114,115,116,117,118,119], whereas oxidized HA hydrogels release thermogenic agents steadily against high-fat-diet-induced obesity [120]. For DKD, HA microneedle patches combine blood glucose control with reactive oxygen species scavenging [136]. Separately, in a clinical bariatric setting, endoscopic submucosal injection of cross-linked HA at the gastroesophageal junction has been evaluated alongside intragastric balloons as a mechanical bulking strategy for obesity [137].

**Figure 2:**
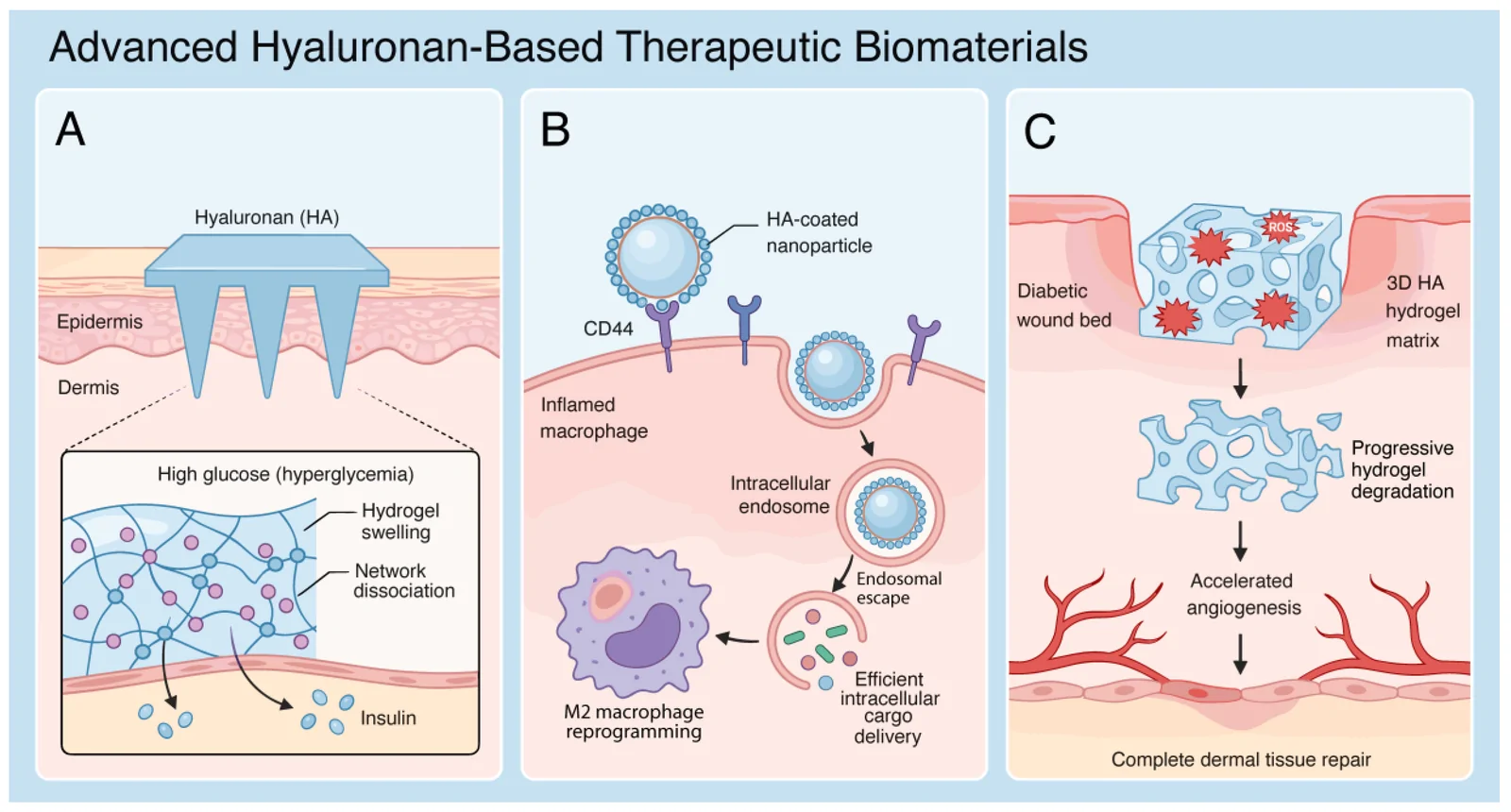
Advanced hyaluronan-based therapeutic biomaterials for metabolic disease management. (**A**) Glucose-responsive dissolving HA-based microneedles penetrate the skin and enable on-demand insulin delivery. In appropriately engineered formulations, glucose-responsive components trigger changes in hydrogel swelling or network integrity under hyperglycemic conditions, promoting insulin release. (**B**) CD44-targeted self-assembled HA-coated nanoparticles selectively bind CD44 on inflamed macrophages, undergoing CD44-mediated endocytosis into intracellular endosomes followed by endosomal escape to achieve efficient intracellular cargo delivery and promote M2 macrophage reprogramming (suppressing inflammatory activation). (**C**) Three-dimensional (3D) HA hydrogel matrices applied to diabetic wound beds actively scavenge reactive oxygen species (ROS); progressive hydrogel degradation over time promotes accelerated angiogenesis and facilitates complete dermal tissue repair. Glucose responsiveness, enhanced ROS-scavenging activity, and specific immunomodulatory effects are formulation-dependent.

CD44-targeted HA nanoparticles and nanoconjugates form a third route. In diet-induced obese and T2D mouse models, self-assembled HA nanoparticles reduced lipid accumulation and improved glucose handling [121,122]. HA nanoparticles home to inflamed intestinal mucosa in colitis [123]. HA-based nanogels carry miR-133a to lessen sepsis-induced cardiomyopathy [124]. HA-bilirubin nanomedicine corrects dysregulated intestinal barrier, gut microbiota, and mucosal immunity [125]. In this class of studies HA functions as a carrier and targeting ligand rather than as the pharmacological target, so the reported benefits do not by themselves demonstrate correction of pathological HA signaling; controls with an empty carrier and with the free drug are required to attribute the effect. These combined HA nanobioconjugates are being tested preclinically for CD44-directed obesity therapy [1]. Exogenous HMW-HA is itself anti-inflammatory in sepsis, allergy, and nerve injury models [52,53,55], which suggests that restoring the HMW-HA-to-LMW-HA ratio, rather than removing HA altogether, may be a sound therapeutic principle [4,9]. CD44-mediated uptake in these studies was shown by carrier accumulation and receptor-dependent internalization in cultured cells and in mouse models, which supports target engagement in those models but does not establish it in patients. Figure 2 depicts advanced hyaluronan-based therapeutic biomaterials and delivery modalities.

**Figure 3:**
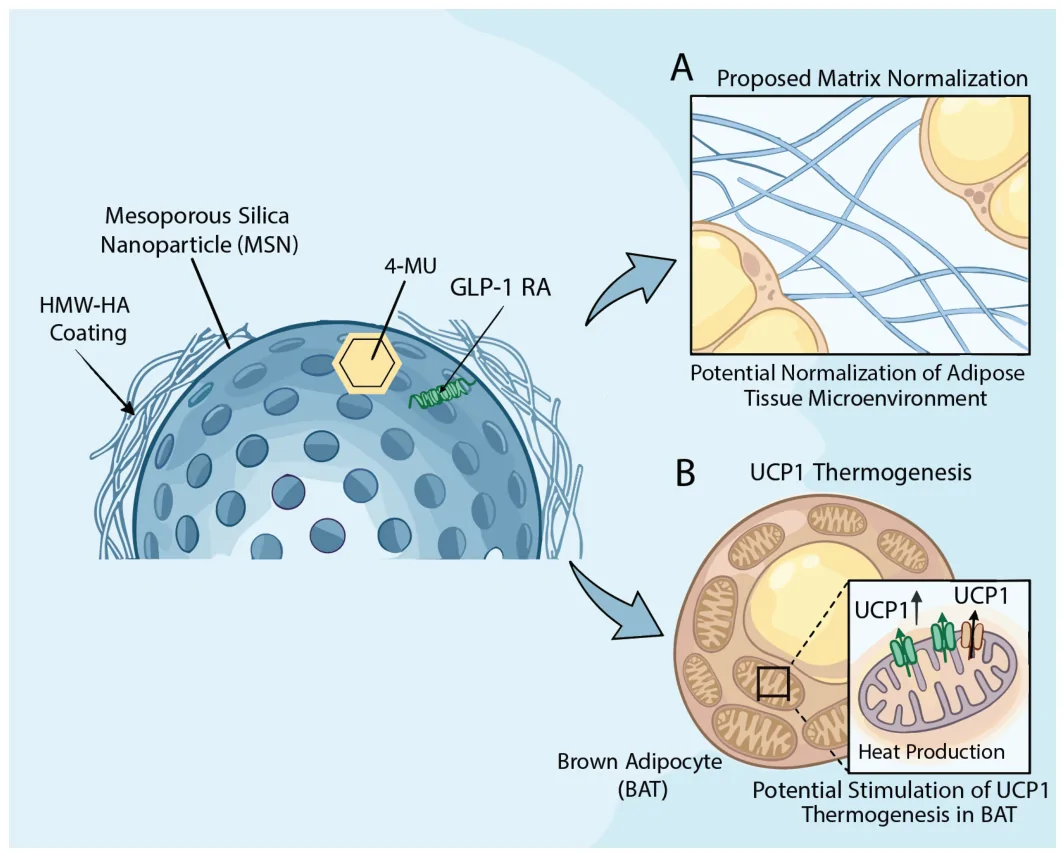
Conceptual design for an HA-based nanoplatform in metabolic disease. Mesoporous silica nanoparticles coated with HMW-HA and co-loaded with 4-MU and GLP-1 receptor agonist peptides are proposed for delivery to adipose tissue. (**A**) The platform could potentially normalize the adipose tissue microenvironment by reducing HA deposition and matrix remodeling. (**B**) The combination might affect UCP1 expression in brown adipocytes, enhancing thermogenesis and energy expenditure. The schematic represents a conceptual framework; the combined formulation remains to be tested experimentally.

From a design standpoint, modulators of the CD44-HA axis built from small molecules, 4-MU, metformin, and CD44 antagonists among them, win on synthesis, bioavailability, and cost but struggle with limited specificity and possible systemic toxicity [1,92]. Nanoconjugates built on HA, by contrast, deliver CD44-targeted drug release with prolonged duration and fewer off-target effects, although scale-up, immunogenicity, and regulatory clearance remain open questions [1,9,95]. Pairing HA synthesis inhibition with CD44-targeted nanocarriers may thus exploit complementary mechanisms and improve metabolic outcomes [1]. The hypothetical outcomes of such a platform are summarized in Figure 3.

### 3.4. Defined-Molecular-Weight Hyaluronan Fragments

Defined-size HA fragments make up a distinct therapeutic category. After oral administration, the 35 kDa fragment HA35 protects the gut, dampens inflammation, and reduces liver injury in diet-induced models [127,128]. A pilot study in healthy adults reported that oral HA35 was well tolerated [129], which is currently the only human safety information for this fragment and does not establish efficacy. Specific HA oligosaccharides also alter fibroblast migration and inflammatory responses depending on their size [58]. For these fragments to reach clinical use, production of defined-molecular-weight preparations that are standardized and endotoxin-free will be essential, and purity should be documented so that endotoxin-related effects are not mistaken for HA fragment activity [17]. Table 3 shows the current clinical development landscape for HA-directed therapies, distinguishing approved agents, repurposed drugs, and preclinical platforms, together with the principal barriers to translation.

### 3.5. Combination Regimens and Repurposing of Approved Drugs

No single HA-directed agent is likely to control a disease as multifactorial as obesity-linked type 2 diabetes, and the fastest route to clinical testing runs through combinations with drugs that already anchor metabolic care. Hymecromone is a candidate for repurposing because it is already prescribed in humans. It primarily limits HA synthesis through UDP-glucuronic acid depletion; changes in HAS expression have also been reported in selected experimental models [86]. Its metabolic actions complement those of incretin-based therapy, since hymecromone raises the thermogenic capacity of brown adipose tissue and improves glycemic control in obese and autoimmune diabetes models [21], whereas GLP-1 receptor agonists act mainly through appetite and insulin secretion [138]. Delivering the peptide with an HA carrier places both mechanisms in one formulation, and HA-modified mesoporous silica nanoparticles have been used for the oral delivery of liraglutide [138]. Metformin provides a second partner: HA-modified carriers concentrate it in CD44-expressing cells in cancer models [139], and injectable HA hydrogels release it in a microenvironment-responsive manner in diabetic wounds [140]. Computational analysis of HA conjugates with anti-diabetic small molecules supports the feasibility of such prodrug designs [141].

Sequencing deserves as much attention as the combination itself. A matrix dense with HA restricts the access of macromolecular drugs, so partial matrix normalization before delivery may improve exposure to the second agent, whereas the reverse order concentrates a drug in CD44-rich tissue before the matrix is remodeled [126]. Early trials will need factorial or sequential designs to separate additive effects from synergistic effects, and the endpoints must capture matrix remodeling alongside glycemic control. Repurposing lowers the regulatory burden because safety data for hymecromone, metformin, and incretin mimetics are extensive [142,143]. Two cautions follow. Combination regimens multiply off-target effects, and the HA-independent actions of hymecromone remain incompletely mapped [93,144]. Benefit observed in one model should also not be projected onto patients without evidence that the target tissue is engaged. Table 4 shows the combinations that are closest to clinical testing. Figure 3 illustrates a proposed nanoplatform that would combine 4-MU and a GLP-1 receptor agonist in a single HMW-HA-coated carrier. This formulation has not been tested experimentally: matrix normalization and UCP1-dependent thermogenesis are outcomes predicted from the separate activities of the two agents [21,138], and should be treated as hypothetical until the co-loaded carrier is characterized in vitro and in vivo.

### 3.6. Patient Selection and Biomarker-Guided Stratification

Whether the right patient receives an agent will matter more than the potency of that agent. HA can be measured in blood, and its concentration and the size distribution of its fragments differ between healthy and metabolically compromised states [26,151]. In obesity-related kidney disease, HA deposition and the abundance of CD44 and RHAMM track tissue injury and predict progression [76,152]. Endothelial glycocalyx markers signal vascular risk in diabetes and critical illness [26,27,153]. A practical stratification strategy therefore pairs a systemic marker with a tissue-specific one, using circulating HA or glycocalyx fragments as the screening layer and imaging- or biopsy-based matrix assessment as the confirmatory layer. Receptor-level information, especially Layilin expression in immune and stromal cells, may complement matrix assessment but has not been standardized for stratification [154]. The framework is proposed rather than validated. Circulating HA is a marker of disease association, and no measurement of HA, of its size distribution, or of glycocalyx fragments has yet been shown prospectively to predict the response to an HA-directed therapy [26,151].

Several requirements follow from that logic. Assays must be calibrated across platforms, because absolute HA values differ between analytical methods and depend on sample handling [26]. Molecular-weight resolution matters when the therapeutic decision depends on whether the axis is dominated by pro-inflammatory fragments or by intact polymer, and size-resolved methods have been validated for surface-attached and soluble HA in methodological settings [155,156]. Early trials should enrich patients with a measurable matrix burden, and endpoints should combine glycemic measures with matrix readouts so that target engagement can be distinguished from symptomatic benefit. Several constraints apply to this framework. Serum HA reflects the balance between production in peripheral tissues and hepatic clearance, so concentrations rise with hepatic dysfunction, systemic inflammation, and age independently of the target tissue, and measured values depend on fasting status, sample handling, and the assay platform [28]. Tissue-level confirmation therefore remains necessary, decision thresholds have not been established for any of these readouts, and distinguishing patients who will respond to a given intervention from those who will not remains an open research question. Table 5 lists candidate markers together with the evidence that supports each of them and the level of that evidence.

### 3.7. Emerging Modalities and Next-Generation Delivery Platforms

Three modalities are advancing faster than the rest. The first is receptor-directed nanomedicine, in which HA-coated carriers accumulate in CD44-rich tissue and deliver anti-inflammatory or metabolic cargo in obesity models [145,159,160]. The second is transdermal delivery, where dissolving microneedles made of HA release insulin, glucagon, or incretin mimetics in response to glucose, and where glucose-responsive and pH-responsive matrices have improved dosing control [146,161,162,163,164]. The third is local therapy for the endocrine pancreas, where islets encapsulated in HA-based matrices retain function and oxygen-releasing hydrogels address the diffusion limit that constrains transplanted islets [165,166]. Local matrices formulated for wound repair follow the same principle and are already being tested in diabetic wounds [147,167,168].

Every modality raises a specific question. For nanomedicine, the issue is whether accumulation reflects target engagement rather than sequestration by the mononuclear phagocyte system [169]. For microneedles, the issue is whether the dose precision and skin tolerability hold up in patients with neuropathy and impaired wound healing [170,171,172,173]. For encapsulation and injectable matrices, the issue is whether biocompatibility persists for months and whether the HA component can be produced at clinical grade without batch-to-batch variation [174]. The same materials are under study for obesity-associated complications, where a hydrogel platform was designed to combine drug delivery with local immunomodulation [175]. A fourth approach, oral delivery of peptides with HA-modified carriers, sits between these categories and has shown favorable pharmacokinetics for liraglutide [138]. Experience with HA products in other indications shows that HA-based materials can clear regulatory review when the indication and the endpoint are defined sharply [107,176]. Beyond metabolic disease, HA-based microneedle–nanocarrier platforms are also being explored for tolerogenic immunotherapy in autoimmune skin disease such as psoriasis [177].

## 4. Challenges and Future Perspectives

The clinical trajectory of PEGPH20 underscores the translational challenges ahead: despite promising preclinical and phase II results, its failure to improve survival in the biomarker-selected phase III HALO-301 trial demonstrates the limits of stratifying patients by total HA abundance alone and highlights the need to manage thromboembolic risk [79,80]. For HA-based biomaterials, scale-up, consistency between batches, immunogenicity, and regulatory paths for drug–device combinations remain open questions, although progress in HA hydrogel engineering has eased some of these barriers [178,179]. HA-directed therapies also face well-defined translational obstacles. Hymecromone already holds clinical approval and is rapidly converted to 4-methylumbelliferyl glucuronide, which itself inhibits HA synthesis, but questions remain about metabolic stability, dose windows, and chronic-use safety for metabolic indications, and liver-related effects, together with several off-target actions, complicate dose selection [93,144]. Countermeasures include patient selection by biomarker, size-specific HA quantification, combining regimens, and adopting rigorous quality-by-design manufacturing.

The HA axis offers a chance to intervene where matrix biology meets metabolism. Several questions come first. Which HAS isoforms and which tissues warrant targeting in T2D and obesity? Does normalization of islet HA restore functional β-cell mass in humans? Is metabolic outcome predicted by HA size distribution instead of total concentration? Can HA-based delivery systems be paired with HA synthesis inhibition to normalize the matrix while releasing drugs in a controlled way? Biomarker-guided patient selection, built on HA profiling by size and matrix signatures tied to CD44, could flag patients most likely to respond to HA-directed therapy [28,50,180]. RNA therapeutics carried by HA, siRNA, microRNA, and shRNA among them, are already being tested in models of metabolic and inflammatory disease [102,115,124], and HA-built platforms that touch immune receptors are being honed for tolerogenic use [75]. Beyond metabolic disease models, sodium hyaluronate-based hydrogels have been used to deliver siRNA for sustained SMAD3 silencing in experimental intrauterine adhesions, providing a further example of HA-based RNA delivery [181]. Further out, gene-editing strategies that adjust HAS or CD44 expression, together with computational drug design, may allow finer targeting of the HA axis, though these approaches remain early-stage. These questions can only be answered through coordinated genetic, pharmacological, and clinical work, yet the emerging evidence casts hyaluronan in the dual role of biomarkers and actionable targets in metabolic medicine.

### 4.1. Positioning Relative to Previous Reviews

Extensive reviews of hyaluronan already exist, so a new synthesis must justify its scope. Strong accounts already cover HA metabolism and function [182], the biology of the polymer in its structural and contextual complexity [183,184], the regulation of HA biosynthesis [185], the coupling of HA synthesis to cellular energy metabolism [186], the role of HA in adipose biology and systemic glucose homeostasis [7,130], and HA in kidney physiology and disease [187]. Therapeutic modulation of HA signaling has also been surveyed [142]. This review differs in three respects. It treats molecular size as an organizing variable and gives the size classes operational definitions, it separates interventions that modify endogenous HA biology from platforms that use HA as a carrier or scaffold, and it concentrates on the translational evidence that links target engagement, stratification, manufacturing, and trial design.

That final point merits emphasis. Most earlier reviews stop at mechanisms, or they catalog delivery systems without tying them to trial design. Section 3 and Section 4 combine the therapeutic landscape, the stratification tools, the manufacturing constraints, and the regulatory questions into a single translational framework, and they spell out the evidence that is still missing. The framework is deliberately falsifiable. If HA accumulation were only a marker of metabolic stress, size-resolved depletion should fail to change insulin sensitivity in adequately powered studies. If HA acts as an amplifier, partial normalization of the matrix should produce measurable benefit even while the primary metabolic insult persists.

### 4.2. Target Engagement and Pharmacodynamic Readouts

A readout showing that the drug reached its target is essential for trials of HA-directed therapy. Circulating HA is an attractive candidate, but it combines production, fragmentation, and hepatic clearance, so a change in concentration cannot be assigned to one tissue [188]. Tissue-level matrix imaging, size-resolved fragment analysis, and receptor-occupancy measurement each answer part of the question [151,155,156]. A practical pharmacodynamic package would pair one systemic marker with one tissue-specific measure and sample at fixed intervals after dosing, because hymecromone is rapidly glucuronidated while its active metabolite contributes to the effect [86,144].

Preclinical studies should report the matrix phenotype that accompanies a metabolic response. Work that shows improved glucose tolerance after HA depletion is most informative when it quantifies HA content, fragment size, and receptor expression in the tissue of interest, and when it separates effects on inflammation from effects on insulin signaling [24,82,88]. Without such measurements, positive and negative results cannot be compared across laboratories, and the field cannot establish which model best predicts human biology. Receptor diversity adds a further layer, because HA receptors beyond CD44 drive distinct pathological processes, with RHAMM mediating metabolic tissue injury [158], and layilin governing immune and cell-motility signaling [154,189].

**Figure 4:**
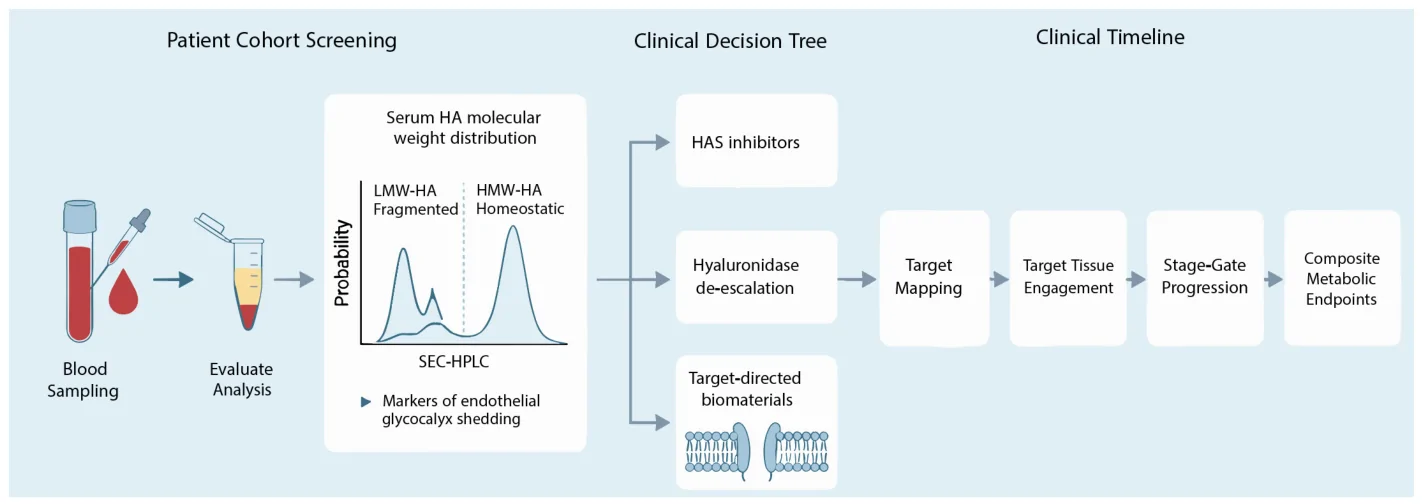
Proposed biomarker-guided stratification and clinical translation framework for HA-directed therapy. (**Left**) Patient cohort screening. Blood sampling followed by SEC-HPLC analysis of serum HA molecular-weight distribution distinguishes patients with fragmented LMW-HA profiles from those with homeostatic HMW-HA profiles. Markers of endothelial glycocalyx shedding are measured in parallel as a vascular risk indicator. (**Middle**) Clinical decision tree. Patients with fragmented HA profiles would be directed to HAS inhibitors, hyaluronidase de-escalation, or target-directed biomaterials, depending on the dominant HA phenotype and tissue involvement. Hyaluronidase de-escalation refers to reducing or stopping enzymatic depletion when size-resolved profiling indicates that the high-molecular-weight pool has been restored; this strategy is hypothetical and would require prospective validation. (**Right**) Clinical timeline. The proposed translational pathway proceeds from target mapping to target tissue engagement, stage-gate progression, and composite metabolic endpoints that combine glycemic control with matrix remodeling readouts. Assay validation, decision thresholds, and the relationship between circulating HA profiles and tissue-specific pathology have not been established.

Figure 4 integrates these pharmacodynamic considerations into a patient-stratification and decision framework, linking serum HA molecular-weight profiling to a clinical decision tree and a staged timeline for target engagement and composite metabolic endpoints. Lipid droplet-targeted fluorescent probes could complement HA-specific measurements by providing exploratory readouts of lipid microenvironment changes in preclinical liver models [190,191]. Their value for evaluating HA-directed interventions remains to be established.

### 4.3. Safety, Immunogenicity, and Manufacturing Considerations

Safety questions cluster around three points. The first concerns the consequences of removing a molecule that carries homeostatic functions, since HMW-HA supports barrier integrity and immune quiescence whereas lower-molecular-weight fragments can promote inflammation depending on local concentration, purity, and tissue context [17,58,192]. While the HALO-301 oncology trial failed despite enriching for hyaluronan-high tumors [79], whether partial, localized HA depletion can achieve a safer and more effective balance in chronic metabolic disease remains untested. The second concerns the immunogenicity of the carrier rather than of the drug, because HA itself is well tolerated while residual protein, cross-linker, or endotoxin can provoke reactions [39,179].

The third point is manufacturing. HA hydrogels and microneedles must be produced with defined molecular weight, defined cross-linking density, and defined limits for residual solvents, because these variables determine both release kinetics and biological response [169,178]. Batch-to-batch consistency is a regulatory requirement for drug–device combinations, and the analytical toolkit resembles the one used for biologics more than the one used for small molecules. Scale-up also has an economic dimension, since the cost of goods for a hydrogel dressing differs by orders of magnitude from that of an oral tablet, and the price must remain defensible against existing standard care [107,193].

### 4.4. Regulatory Pathways and Trial Endpoints

Repurposed small molecules and HA-based devices follow different regulatory routes. Hymecromone already has a clinical dossier, so an additional metabolic indication can be pursued if dose, exposure, and the safety database are adequate for chronic use [93,144]. HA devices enter through the device or combination-product route, where the principal evidence requirements concern biocompatibility, degradation, and reproducibility of manufacturing [169,178]. Biomarker claims sit between the two, because a marker used for stratification is not the same as a marker used for monitoring, and the analytical validation package differs accordingly [26,151].

Endpoint selection will decide whether trials can succeed. Glycated hemoglobin and fasting glucose remain the regulatory currency, but they change slowly and they do not report matrix biology. Composite endpoints that combine a glycemic measure with a matrix readout, a wound-closure measure, or a vascular function measure are more informative, provided that each component is prespecified, is clinically meaningful, is measured with a validated assay, and is analyzed independently; combining glycemic and matrix measures does not by itself improve the interpretation of a trial [151,193]. Early-phase studies should also preselect patients with a measurable HA burden, because a trial that enrolls unstratified participants dilutes any real effect and cannot distinguish a failed mechanism from an insensitive design [7,76]. Randomized experience in diabetic foot ulcers illustrates both the feasibility of such designs and the difficulty of demonstrating benefit beyond optimized standard care [107].

### 4.5. Priority Research Agenda

A small set of unresolved questions defines the near-term agenda. Target selection remains open, namely which HAS isoform should be engaged in each metabolic tissue and whether isoform-selective inhibitors can achieve an acceptable safety margin [92,185]. Measurement remains open, namely whether size-resolved HA quantification predicts therapeutic response better than total concentration [155,156]. Combination remains open, namely whether hymecromone and incretin-based therapy act additively or synergistically in patients [21,138]. Delivery remains open, namely whether receptor-directed platforms achieve functional target engagement in adipose tissue and islets rather than passive accumulation [145,165]. Manufacturing remains open, namely which analytical and process controls are sufficient to guarantee batch consistency for HA devices [178,179].

## 5. Conclusions

Once viewed as a passive matrix component, hyaluronan has become a therapeutic target in metabolic disease, but that recognition has not yet produced clinical success. The evidence reviewed here points in one direction. Molecular size is one important determinant of whether HA protects or harms metabolic tissue, although concentration, preparation purity, receptor context, and tissue location modify the response. Systemic depletion has not yet produced a survival benefit, most clearly in the phase III HALO-301 trial, in which patients were selected for hyaluronan-high tumors; that result shows the limits of depletion in oncology and does not establish molecular size as the explanation. Restoring the balance between HA synthesis and degradation, not eliminating the molecule, is the therapeutic objective. Reaching it will require size-resolved quantification, validated stratification markers, and trial designs that measure matrix remodeling alongside glycemic control. The near-term opportunity lies in combining approved agents, such as hymecromone, with incretin-based therapy, while CD44-targeted nanocarriers, glucose-responsive microneedles, and encapsulated islet platforms mature in parallel. Three categories of evidence should be separated. Demonstrated in preclinical models: HA accumulates in metabolically injured tissue; genetic or pharmacological manipulation of HA synthesis or CD44 changes insulin sensitivity and inflammation and defined-size fragments such as HA35 modify gut and liver injury. Established in humans: HA is measurable in blood and associates with hepatic, renal, and vascular disease; hymecromone is an approved drug with an acceptable safety profile for its current indication, and PEGPH20 does not improve survival in hyaluronan-high pancreatic cancer. Still hypothetical: Size-resolved profiling as a predictive biomarker of treatment response, combination regimens such as hymecromone plus incretin therapy, receptor-directed nanocarriers, and glucose-responsive microneedles can be treatments for metabolic disease. HA is best understood as an amplifier of metabolic stress. Whether that amplifier can be turned down in patients rather than silenced in model systems is the question that will define the next phase of this field.

## Figures and Tables

**Table 1:** Molecular-weight classes of hyaluronan: operational definitions, origin, and reported activity.

Size Class	Operational Size Range	Typical Origin or Preparation	Receptor Engagement	Reported Biological Activity	Caveats	Refs
HA oligosaccharides	About ten saccharides or fewer (roughly up to 2 kDa)	Enzymatic digestion or synthesis	CD44 with monovalent binding; TLR2/4	Pro-inflammatory signaling in some preparations	Depends strongly on concentration and purity	[16,48,58]
Low-molecular-weight HA (small fragments)	About 10–100 kDa	Hyaluronidase or oxidative cleavage during inflammation	CD44, TLR2/4, RHAMM	Cytokine induction, angiogenesis, and macrophage activation in many models	Endotoxin contamination and dose confound interpretation	[14,16,17,43,56]
Intermediate-molecular-weight HA	About 100–1000 kDa	Partial degradation of the native polymer	Multivalent CD44 engagement	Mixed and context-dependent effects	Rarely reported as a separate class	[16,58]
High-molecular-weight HA	Above about 1000 kDa	Native matrix; products of HAS1 and HAS2	Multivalent CD44 engagement	Hydration, barrier support, and immunosuppression in most contexts	Can be pro-inflammatory at high concentration or with specific binding proteins	[16,35,56,58]
Non-enzymatic fragmentation	Not size-defined	Reactive oxygen species and transition-metal chemistry	Products feed the classes above	Converts the protective polymer into smaller signaling-active fragments	Difficult to separate from enzymatic cleavage in vivo	[14,43]

**Table 2:** Hyaluronan metabolic nodes as therapeutic targets: druggability and intervention status.

Component	Class	Principal Metabolic Function	Druggability/Intervention Status	Refs
HAS1	HA synthase	Maintains HMW-HA; anti-inflammatory	No selective inhibitor is available. HAS1 is generally tissue-protective, so the therapeutic aim is to inhibit HAS2 and HAS3 while sparing HAS1; this is an indirect strategy that has not been tested.	[35]
HAS2	HA synthase	Major regulated synthase; drives islet, liver, and adipose HA accumulation	Indirect pharmacological modulation. 4-MU suppresses HAS2 transcription and is the only inhibitor with clinical experience; no HAS2-selective compound has been validated in humans, so isoform selectivity remains an unmet need.	[12,20,31,78]
HAS3	HA synthase	Generates LMW-HA; implicated in context-dependent inflammatory tissue injury	Direct target. No selective inhibitor is available; HAS3 silencing reduces injury in preclinical models, which is indirect evidence of druggability.	[36]
HYAL1/2, PH20	Hyaluronidases	Degrade HA	Exogenous enzyme replacement/addition for HA degradation. PEGPH20 has been evaluated in phase II and III trials for HA depletion: HALO-202 improved progression-free survival in hyaluronan-high tumors, whereas HALO-301 showed no survival benefit.	[23,39,79,80]
Reactive oxygen species/transition metals	Non-enzymatic degradative pathway	Fragment HMW-HA into signaling-active LMW-HA	Indirect target. No HA-specific inhibitor is available; antioxidant strategies such as superoxide dismutase 3 reduce oxidative fragmentation.	[14,43]
TMEM2, KIAA1199/CEMIP	Extracellular HA depolymerases	Generate size-diverse fragments	Direct target. No selective inhibitor is available; TMEM2 and CEMIP remain preclinical targets.	[40,41]
AMPK	Energy-sensing kinase	Inhibits HAS2 activity	Indirect modulator. AMPK inhibits HAS2 activity, but AMPK activators such as metformin are not HA-specific and their effects cannot be attributed to HA alone.	[34]
CD44	HA receptor	Required for HA-dependent insulin resistance	Direct target. Anti-CD44 monoclonal antibodies prevent diabetes in non-obese diabetic mice, and CD44 deletion improves insulin sensitivity in high-fat-fed mice; CD44-targeted nanocarriers remain preclinical. No CD44-directed agent has entered metabolic trials.	[1,24,59]
RHAMM/HMMR	HA receptor	Mediates obesity-related kidney injury signaling	Direct target. No selective inhibitor is available; genetic or pharmacological RHAMM blockade reduces obesity-related kidney injury in preclinical models.	[76]
TLR2/4	Pattern-recognition receptors	Participate in context- and purity-dependent LMW-HA inflammatory signaling	Indirect target. No HA-specific inhibitor is available; downstream TAK-1 and NF-κB signaling can be blocked, but such inhibition is not selective for HA fragments.	[13,15]

**Table 3:** Hyaluronan-based therapeutic strategies in metabolic disease: designs, applications, advantages, and limitations, with the development stage and evidence level as reported in the studies.

Strategy	Category	Design/ Mechanism	Application	Stage	Advantages	Limitations	Refs
4-MU (hymecromone)	Endogenous HA modulation	Inhibits HA synthesis; active metabolite 4-MUG contributes	T2D, obesity, diabetic kidney disease, islet transplantation	Preclinical; approved drug repurposing	Oral bioavailability; cost-effective; clinically approved	Off-target effects; limited specificity; dose optimization	[20,21,22,86,87,88,89,90,91,92,93]
PEGPH20	Endogenous HA modulation	Pegylated hyaluronidase degrades tissue HA	Insulin resistance, obesity, fibrotic disease	Preclinical in metabolic disease; completed phase II and III trials in oncology	Potent enzymatic HA depletion; rapidly reversible	Thromboembolic risk; patient stratification; phase III failure in oncology	[23,25,39,79,80,82,94]
HA-based hydrogels	HA carrier/scaffold	Wound dressings with ROS scavenging, antimicrobial, and drug/exosome delivery	Diabetic wounds	Preclinical; HA dressings are commercially available and have been evaluated in clinical trials of diabetic foot ulcers	Biocompatible; glucose-responsive; accelerates healing	Batch-to-batch consistency; cost-effectiveness	[95,96,97,98,99,100,101,102,103,104,105,106,107]
HA microneedles (insulin)	HA carrier/scaffold	Dissolving arrays; glucose-responsive or hypoxia-responsive release	Diabetes	Preclinical	Painless; glucose-responsive closed-loop delivery	Scalability; regulatory pathway	[108,109,110,111,112,113]
HA microneedles (obesity)	HA carrier/scaffold	Delivery of liraglutide, semaglutide, oleanolic acid, hesperidin; photothermal fat browning	Obesity	Preclinical	Non-invasive; sustained delivery; patient-friendly	Immunogenicity; batch consistency	[114,115,116,117,118,119,120]
HA nanoparticles/nanoconjugates	HA carrier/scaffold	CD44-targeted self-assembled systems	T2D, obesity	Preclinical	High specificity via CD44 targeting; prolonged release; reduced off-target effects	Manufacturing complexity; immunogenicity; regulatory hurdles	[121,122,123,124,125,126]
HA35	Endogenous HA modulation	Defined 35 kDa HA fragment, oral administration	Metabolic-associated liver disease	Preclinical; one pilot safety study in healthy adults	Orally active; gut-protective and anti-inflammatory	Defined-MW production; endotoxin-free standardization	[127,128,129]
Exogenous HMW-HA	Endogenous HA modulation	Anti-inflammatory/immunosuppressive polymer	Sepsis, allergy, nerve injury	Preclinical	Anti-inflammatory; immunosuppressive in most contexts	Context-dependent; can be pro-inflammatory under specific conditions	[52,53,55]

**Table 4:** Combination and repurpose strategies for HA-directed therapy, with the mechanistic rationale, development stage, evidence that the combination itself has been tested, and principal risks of each approach.

Strategy or Combination	Mechanism and Target	Metabolic Context	Development Stage	Main Opportunity	Principal Risk	Evidence	Refs
Hymecromone plus GLP-1 receptor agonist	Reduced HA synthesis with incretin signaling	Obesity with type 2 diabetes	Proposed; each agent is approved separately but the combination has not been tested	Fast repurposing path	Additive gastrointestinal effects	Not tested together	[21,86,138]
Hymecromone plus metformin	Matrix normalization with better insulin action	Type 2 diabetes	Proposed; single-agent data only	Low cost with existing safety data	Limited benefit at low HA burden	Not tested together	[139,140,144]
CD44 blockade plus insulin sensitizer	Interruption of HA-CD44 signaling	Insulin resistance	Preclinical; single-agent data only	Targets the receptor arm of the axis	Effects of sustained CD44 blockade	Not tested together	[24,143]
HA nanoparticle plus anti-inflammatory cargo	CD44-directed tissue accumulation	Obesity and colitis	Preclinical	Tissue-selective delivery	Batch consistency and clearance	Tested as a single formulation	[122,145]
HA microneedle plus insulin or glucagon	Transdermal glucose-responsive dosing	Type 1 and type 2 diabetes	Preclinical	Needle-free closed-loop dosing	Skin tolerability and dose precision	Tested as a single formulation	[110,113,146]
HA hydrogel plus antioxidant or exosome cargo	Wound-bed conditioning with sustained release	Diabetic wounds	Preclinical; HA dressings alone are in clinical use	Addresses an unmet complication	Device-combination regulatory path	Tested as a single formulation	[147,148,149,150]
Enzymatic HA depletion plus cytotoxic therapy	Matrix barrier reduction before exposure	Oncology, transferable lesson	Phase III (HALO-301): no overall survival benefit	Defines stratification needs	Thromboembolic risk without benefit	Tested together in phase II and III trials	[79,80]
Defined-size fragments plus standard care	Barrier and anti-inflammatory tone	Gut–liver axis and liver disease	Preclinical (HA35 monotherapy)	Oral dosing with good tolerability	Dose finding and standardization	Not tested together	[127,128]

**Table 5:** Candidate biomarkers and stratification tools for HA-directed therapy, with the biological meaning, evidence, and practical limitations of each readout, distinguishing markers associated with disease from markers shown to predict treatment response.

Readout.	Specimen	Biological Meaning	Evidence in Metabolic Disease	Practical Considerations	Evidence Level	Refs
Total HA concentration	Serum or plasma	Overall HA burden and clearance	Elevated in insulin resistance, liver disease, kidney disease, and critical illness	Absolute values depend on the assay platform	Disease-associated; not demonstrated to predict treatment response	[7,28]
HA molecular-weight profile	Serum or tissue extract	Intact polymer versus fragments	Size-dependent signaling in adipose and vascular tissue	Needs a standardized size-resolved assay	Disease-associated; predictive value untested	[151,155,156]
Glycocalyx degradation products	Plasma	Endothelial injury and permeability	Predicts vascular complications and mortality in critical illness	Fragments degrade quickly after sampling	Prognostic in critical illness; not treatment-predictive	[26,27,153]
CD44 abundance	Adipose or kidney biopsy	Receptor arm of the axis	Associated with tissue inflammation and insulin resistance	Invasive sampling limits routine use	Tissue-level association; not clinically validated	[76,157]
RHAMM abundance	Tissue or circulating cells	Alternative HA receptor signaling	Linked to kidney injury and hepatic oxidative stress	Assay availability remains limited	Preclinical association only	[76,158]
HAS isoform transcripts	Adipose or islet tissue	Synthetic capacity of the tissue	HAS2 expression tracks matrix expansion in obesity	Requires access to tissue	Mechanistic and preclinical only	[7,21,22]

## Data Availability

No new data were created or analyzed during this study. Data sharing is not applicable to the review.

## Supplementary Materials

The following supporting information can be downloaded at https://www.mdpi.com/article/10.3390/ijms27219712/s1.

## Abbreviations

**HA**	hyaluronic acid (hyaluronan)
**HMW-HA**	high-molecular-weight hyaluronan
**LMW-HA**	low-molecular-weight hyaluronan
**HAS2**	hyaluronan synthase 2
**HYAL1/2**	hyaluronidases 1 and 2
**4-MU**	4-methylumbelliferone
**4-MUG**	4-methylumbelliferyl glucuronide
**CD44**	cluster of differentiation 44
**RHAMM**	receptor for hyaluronan-mediated motility
**TLR**	Toll-like receptor
**ECM**	extracellular matrix
**T2D**	type 2 diabetes
**DIO**	diet-induced obesity
**BAT**	brown adipose tissue
**UCP1**	uncoupling protein 1
**PEGPH20**	pegylated recombinant human hyaluronidase PH20
**DKD**	diabetic kidney disease
**IR**	insulin resistance
**ROS**	reactive oxygen species
**NF-κB**	nuclear factor kappa B
**LPS**	lipopolysaccharide
**GLP-1**	glucagon-like peptide-1
**DAMPs**	damage-associated molecular patterns
**TAK-1**	transforming growth factor beta-activated kinase 1
**p38-MAPK**	p38 mitogen-activated protein kinase
**TNF-α**	tumor necrosis factor-alpha
**UDP**	uridine diphosphate
**PPAR-**γ	peroxisome proliferator-activated receptor gamma
**AMPK**	AMP-activated protein kinase
