# Supplementary material for: Emerging Therapeutic Strategies for Targeting Hyaluronan in Metabolic Disease from Molecular Design to Clinical Translation
Source: Int J Mol Sci. 2026 Oct 29;27(21):9712. doi: 10.3390/ijms27219712 (PMC13763286; doi:10.3390/ijms27219712)
Supplement: Supplementary file 1 [file ijms-27-09712-s001.zip › ijms-4601681-supplementary.pdf]

Search Strategy and Study Selection

The literature search was performed in PubMed using the strategy shown in Table S1, and the last search run was on September 1, 2026. The search combined three concept blocks: hyaluronan-related terms, metabolic disease-related terms, and molecular target/therapeutic terms, yielding 1,478 records. The combined search strategy is as follows.

Table S1. Search strategy and records retrieved from PubMed.

		Records
#1	("hyaluronan"[Title/Abstract] OR "hyaluronic acid"[Title/Abstract] OR "HMW-HA"[Title/Abstract] OR "LMW-HA"[Title/Abstract])	40,923
#2	("metabolic disease"[Title/Abstract] OR "metabolic syndrome"[Title/Abstract] OR "obesity"[Title/Abstract] OR "diabetes"[Title/Abstract] OR "type 2 diabetes"[Title/Abstract] OR "insulin resistance"[Title/Abstract] OR "adipose tissue"[Title/Abstract] OR "diabetic kidney disease"[Title/Abstract] OR "diabetic wound"[Title/Abstract] OR "wound healing"[Title/Abstract] OR "islet"[Title/Abstract] OR "beta cell"[Title/Abstract])	1,248,886
#3	("HAS1"[Title/Abstract] OR "HAS2"[Title/Abstract] OR "HAS3"[Title/Abstract] OR "hyaluronan synthase"[Title/Abstract] OR "hyaluronidase"[Title/Abstract] OR "CD44"[Title/Abstract] OR "RHAMM"[Title/Abstract] OR "TLR2"[Title/Abstract] OR "TLR4"[Title/Abstract] OR "4-methylumbelliferone"[Title/Abstract] OR "4-MU"[Title/Abstract] OR "hymecromone"[Title/Abstract] OR "PEGPH20"[Title/Abstract] OR "hydrogel"[Title/Abstract] OR "microneedle"[Title/Abstract] OR "nanoparticle"[Title/Abstract] OR "biomarker"[Title/Abstract] OR "stratification"[Title/Abstract])	715,717
Total	#1 & #2 & #3	1,478

("hyaluronan"[Title/Abstract] OR "hyaluronic acid"[Title/Abstract] OR "HMW-HA"[Title/Abstract] OR "LMW-HA"[Title/Abstract]) AND

("metabolic disease"[Title/Abstract] OR "metabolic syndrome"[Title/Abstract] OR "obesity"[Title/Abstract] OR "diabetes"[Title/Abstract] OR "type 2 diabetes"[Title/Abstract] OR "insulin resistance"[Title/Abstract] OR "adipose tissue"[Title/Abstract] OR "diabetic kidney disease"[Title/Abstract] OR "diabetic wound"[Title/Abstract] OR "wound healing"[Title/Abstract] OR "islet"[Title/Abstract] OR "beta cell"[Title/Abstract])
AND ("HAS1"[Title/Abstract] OR "HAS2"[Title/Abstract] OR "HAS3"[Title/Abstract] OR "hyaluronan synthase"[Title/Abstract] OR "hyaluronidase"[Title/Abstract] OR "CD44"[Title/Abstract] OR "RHAMM"[Title/Abstract] OR "TLR2"[Title/Abstract] OR "TLR4"[Title/Abstract] OR "4-methylumbelliferone"[Title/Abstract] OR "4-MU"[Title/Abstract] OR "hymecromone"[Title/Abstract] OR "PEGPH20"[Title/Abstract] OR "hydrogel"[Title/Abstract] OR "microneedle"[Title/Abstract] OR "nanoparticle"[Title/Abstract] OR "biomarker"[Title/Abstract] OR "stratification"[Title/Abstract])

After excluding specific publication types (comment, case reports, conference abstracts, congress materials, letters, and editorials), 1,473 records remained. A further 9 records were excluded because of language (Chinese, n = 6; French, n = 1; other, n = 2), and 3 preprints were excluded, leaving 1,461 records. Of these, 872 full-text articles were assessed for eligibility, and 193 references were finally included in the review (Table S2).

Table S2. Screening and selection of records.

Exclude		Records
Article Type	comment[pt] = 1<br>case reports[pt] = 4<br>conference abstract[pt] = 0<br>congress[pt] = 0<br>letter[pt] = 0<br>editorial[pt] = 0	1,473
Language	Chinese=6<br>French=1<br>Other=2	1,464
Preprints		1,461
Full-text articles assessed for eligibility		872
References included in the review		193

Temporal Distribution of Retrieved Publications

A total of 1478 records were initially retrieved, with 1461 records remaining after the exclusion of specific publication types, non-English articles, and preprints. Output remained low before 2000, increased steadily thereafter, and accelerated markedly after 2015, highlighting the rapid growth and promising future of this field.

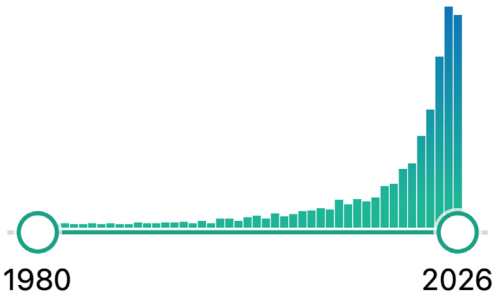

Figure S1. Annual distribution of PubMed-retrieved publications on hyaluronan in metabolic disease

Human hyaluronidases and HA-depolymerizing enzymes

The major routes of HA turnover in humans, including six hyaluronidase-like genes, two non-canonical HA depolymerases, and non-enzymatic cleavage, are summarized in Table S3. These enzymes differ in localization, substrate preference, and the fragment sizes they generate. HYAL1 is the principal lysosomal hyaluronidase, whereas HYAL2 initiates extracellular cleavage of HMW-HA at the cell surface. HYAL3 has little or no catalytic activity toward HA, and HYAL4 acts on chondroitin sulfate rather than HA. PH20 (SPAM1) is the testicular enzyme that provides the therapeutic agent PEGPH20. TMEM2 (CEMIP2) and CEMIP (KIAA1199) depolymerize HA through non-canonical routes, and reactive oxygen species and transition metals fragment HA without enzymatic activity. Together, these routes determine the size distribution of HA and its biological activity.

Table S3. Human hyaluronidases and hyaluronic acid depolymerizing enzymes.

Gene or protein	Class and localization	Substrate and products	Metabolic relevance	Refs
HYAL1	Hyaluronidase; lysosomal, also secreted	Degrades HA oligosaccharides to tetrasaccharides	Systemic HA catabolism; loss of HYAL1 alters endothelial glycocalyx integrity in diabetic models	[1,2]
HYAL2	Hyaluronidase; glycosylphosphatidylinositol-anchored and associated with CD44	Initiates extracellular cleavage of HMW-HA	First step in generating smaller fragments, including potentially signaling-active or context-dependent pro-inflammatory species	[1,2]
HYAL3	Hyaluronidase-like protein; intracellular	Little or no catalytic activity toward HA	Function uncertain; may regulate HYAL1 and HYAL2 activity	[1,2]
HYAL4	Chondroitinase; intracellular	Acts on chondroitin sulfate rather than HA	Shows that this gene family is not exclusively directed at HA	[1]
PH20 (SPAM1)	Hyaluronidase; testicular and acrosomal	Cleaves HMW-HA; source of the therapeutic enzyme PEGPH20	Basis of enzymatic depletion strategies in oncology	[1,3-5]
TMEM2 (CEMIP2)	Cell-surface HA depolymerase	Produces size-diverse fragments at the cell surface	Clears pericellular HA and modulates receptor availability	[6]
CEMIP (KIAA1199)	Secreted or cell-associated HA depolymerase	Non-canonical cleavage of HA	Expressed in inflamed and fibrotic tissue and contributes to fragment generation	[7]
Non-enzymatic cleavage	Reactive oxygen species and transition metals	Fragments HMW-HA without enzyme activity	Relevant in inflamed and metabolically stressed tissue	[8,9]

Representative studies of hyaluronan-directed interventions

Preclinical and clinical investigations evaluating hyaluronan-directed therapies encompass pharmacological synthesis inhibition, enzymatic degradation, biomaterial-based delivery systems, and defined-molecular-weight fragments, as summarized in Table S4. These studies reflect diverse disease models, administration routes, and molecular endpoints, highlighting therapeutic efficacy across metabolic and oncological settings as well as the principal translational limitations encountered in current experimental designs.

Table S4. Representative studies of HA-directed interventions, with model, route and dose, principal outcome, and principal limitation.

Study	Model or population	Intervention, route, and dose	Principal outcome	Principal limitation	Refs
Nagy et al., 2023	db/db mice; human cadaveric islets	4-MU, oral, and CD44 deletion (dosing in the primary report)	Preserved glycemic control and insulin concentrations despite weight gain; reduced beta-cell apoptosis	Single model; HA-independent effects of 4-MU not excluded	[10]
Nagy et al., 2015	Non-obese diabetic mice (autoimmune insulitis)	4-MU, oral (dosing in the primary report)	Prevented progression to diabetes and preserved beta-cell mass	Autoimmune model whose mechanism differs from metabolic type 2 diabetes	[11]
Grandoch et al., 2019	Mice on a diabetogenic diet	4-MU, oral, and Has2/Has3 deletion (dosing in the primary report)	Increased brown adipose tissue thermogenesis and UCP1 expression; improved glucose homeostasis; reduced weight gain	Pharmacological and genetic effects not separated; 4-MU is not HA-specific	[12]
Kang et al., 2013	High-fat-fed diet-induced obese mice	PEGPH20, systemic	Reduced muscle HA, fat mass, and adipocyte size; reversed hepatic and muscle insulin resistance	Acute treatment; long-term safety not established	[13]
Hasib et al., 2019	CD44-deficient and wild-type mice on a 60% high-fat diet for 16 weeks	Genetic CD44 deletion with or without PEGPH20; no drug dose applies	CD44 deletion preserved muscle insulin sensitivity, and PEGPH20 no longer improved it	Germline knockout; developmental compensation cannot be excluded	[14]
Ray et al., 2025	Mice on a high-fat, fructose, and cholesterol diet	HA35, oral gavage, 15 mg/kg daily for 6 days	Reduced hepatocyte injury, inflammatory gene expression, and fibrosis markers	Short treatment; a single sex; a single model	[15]
Bellar et al., 2019	Healthy human volunteers	HA35, oral, 140 mg once daily for 7 days	Well tolerated with no safety signal; gut permeability and microbiota were exploratory endpoints	Pilot study in healthy participants without an efficacy endpoint	[16]
Yu et al., 2015	Streptozotocin-induced diabetic mice	Glucose-responsive HA microneedle patch, transdermal	Fast glucose-responsive insulin release with improved glycemic control	Preclinical; batch consistency and skin tolerability not established	[17]
Hingorani et al., 2018	Phase II trial in metastatic pancreatic cancer (n = 279)	PEGPH20 plus nab-paclitaxel and gemcitabine, intravenous	Improved progression-free survival overall and in hyaluronan-high tumors; higher objective response rate	Subgroup analysis; thromboembolic events; no overall survival benefit	[5]
Van Cutsem et al., 2020	Phase III trial in hyaluronan-high metastatic pancreatic cancer (n = 494)	PEGPH20 3.0 microgram/kg twice weekly plus nab-paclitaxel 125 mg/m2 and gemcitabine 1,000 mg/m2, intravenous	Higher objective response rate but no improvement in overall or progression-free survival	Oncology population without metabolic endpoints; depletion-only strategy	[4]

References

1. Csoka, A.B.; Frost, G.I.; Stern, R. The six hyaluronidase-like genes in the human and mouse genomes. *Matrix Biol* **2001**, *20*, 499-508, doi:10.1016/s0945-053x(01)00172-x.
2. Stern, R.; Kogan, G.; Jedrzejas, M.J.; Soltés, L. The many ways to cleave hyaluronan. *Biotechnol Adv* **2007**, *25*, 537-557, doi:10.1016/j.biotechadv.2007.07.001.
3. Huang, Z.; Zhao, C.; Chen, Y.; Cowell, J.A.; Wei, G.; Kultti, A.; Huang, L.; Thompson, C.B.; Rosengren, S.; Frost, G.I.; et al. Recombinant human hyaluronidase PH20 does not stimulate an acute inflammatory response and inhibits lipopolysaccharide-induced neutrophil recruitment in the air pouch model of inflammation. *J Immunol* **2014**, *192*, 5285-5295, doi:10.4049/jimmunol.1303060.
4. Van Cutsem, E.; Tempero, M.A.; Sigal, D.; Oh, D.Y.; Fazio, N.; Macarulla, T.; Hitre, E.; Hammel, P.; Hendifar, A.E.; Bates, S.E.; et al. Randomized Phase III Trial of Pegvorhyaluronidase Alfa With Nab-Paclitaxel Plus Gemcitabine for Patients With Hyaluronan-High Metastatic Pancreatic Adenocarcinoma. *J Clin Oncol* **2020**, *38*, 3185-3194, doi:10.1200/jco.20.00590.
5. Hingorani, S.R.; Zheng, L.; Bullock, A.J.; Seery, T.E.; Harris, W.P.; Sigal, D.S.; Braiteh, F.; Ritch, P.S.; Zalupski, M.M.; Bahary, N.; et al. HALO 202: Randomized Phase II Study of PEGPH20 Plus Nab-Paclitaxel/Gemcitabine Versus Nab-Paclitaxel/Gemcitabine in Patients With Untreated, Metastatic Pancreatic Ductal Adenocarcinoma. *J Clin Oncol* **2018**, *36*, 359-366, doi:10.1200/jco.2017.74.9564.
6. Yamamoto, H.; Tobisawa, Y.; Inubushi, T.; Irie, F.; Ohyama, C.; Yamaguchi, Y. A mammalian homolog of the zebrafish transmembrane protein 2 (TMEM2) is the long-sought-after cell-surface hyaluronidase. *J Biol Chem* **2017**, *292*, 7304-7313, doi:10.1074/jbc.M116.770149.
7. Yoshida, H.; Nagaoka, A.; Kusaka-Kikushima, A.; Tobiishi, M.; Kawabata, K.; Sayo, T.; Sakai, S.; Sugiyama, Y.; Enomoto, H.; Okada, Y.; et al. KIAA1199, a deafness gene of unknown function, is a new hyaluronan binding protein involved in hyaluronan depolymerization. *Proc Natl Acad Sci U S A* **2013**, *110*, 5612-5617, doi:10.1073/pnas.1215432110.
8. Soltés, L.; Kogan, G. Catabolism of hyaluronan: involvement of transition metals. *Interdiscip Toxicol* **2009**, *2*, 229-238, doi:10.2478/v10102-009-0026-y.
9. Kwon, M.J.; Han, J.; Kim, B.H.; Lee, Y.S.; Kim, T.Y. Superoxide dismutase 3 suppresses hyaluronic acid fragments mediated skin inflammation by inhibition of toll-like receptor 4 signaling pathway: superoxide dismutase 3 inhibits reactive oxygen species-induced trafficking of toll-like receptor 4 to lipid rafts. *Antioxid Redox Signal* **2012**, *16*, 297-313, doi:10.1089/ars.2011.4066.
10. Nagy, N.; Kaber, G.; Sunkari, V.G.; Marshall, P.L.; Hargil, A.; Kuipers, H.F.; Ishak, H.D.; Bogdani, M.; Hull, R.L.; Grandoch, M.; et al. Inhibition of hyaluronan synthesis prevents β-cell loss in obesity-associated type 2 diabetes. *Matrix Biol* **2023**, *123*, 34-47, doi:10.1016/j.matbio.2023.09.003.
11. Nagy, N.; Kaber, G.; Johnson, P.Y.; Gebe, J.A.; Preisinger, A.; Falk, B.A.; Sunkari, V.G.; Gooden, M.D.; Vernon, R.B.; Bogdani, M.; et al. Inhibition of hyaluronan synthesis restores immune tolerance during autoimmune insulitis. *J Clin Invest* **2015**, *125*, 3928-3940, doi:10.1172/jci79271.
12. Grandoch, M.; Flögel, U.; Virtue, S.; Maier, J.K.; Jelenik, T.; Kohlmorgen, C.; Feldmann, K.; Ostendorf, Y.; Castañeda, T.R.; Zhou, Z.; et al. 4-Methylumbelliferone improves the

thermogenic capacity of brown adipose tissue. *Nat Metab* **2019**, *1*, 546-559, doi:10.1038/s42255-019-0055-6.
13. Kang, L.; Lantier, L.; Kennedy, A.; Bonner, J.S.; Mayes, W.H.; Bracy, D.P.; Bookbinder, L.H.; Hasty, A.H.; Thompson, C.B.; Wasserman, D.H. Hyaluronan accumulates with high-fat feeding and contributes to insulin resistance. *Diabetes* **2013**, *62*, 1888-1896, doi:10.2337/db12-1502.
14. Hasib, A.; Hennayake, C.K.; Bracy, D.P.; Bugler-Lamb, A.R.; Lantier, L.; Khan, F.; Ashford, M.L.J.; McCrimmon, R.J.; Wasserman, D.H.; Kang, L. CD44 contributes to hyaluronan-mediated insulin resistance in skeletal muscle of high-fat-fed C57BL/6 mice. *Am J Physiol Endocrinol Metab* **2019**, *317*, E973-e983, doi:10.1152/ajpendo.00215.2019.
15. Ray, S.; Huang, E.; McMullen, M.R.; Jatana, S.; de la Motte, C.; Nagy, L.E. 35k Da specific-sized hyaluronan ameliorates high-fat diet-induced liver injury in murine model of moderate obesity. *Matrix Biol* **2025**, *136*, 1-8, doi:10.1016/j.matbio.2024.12.010.
16. Bellar, A.; Kessler, S.P.; Obery, D.R.; Sangwan, N.; Welch, N.; Nagy, L.E.; Dasarathy, S.; de la Motte, C. Safety of Hyaluronan 35 in Healthy Human Subjects: A Pilot Study. *Nutrients* **2019**, *11*, doi:10.3390/nu11051135.
17. Yu, J.; Zhang, Y.; Ye, Y.; DiSanto, R.; Sun, W.; Ranson, D.; Ligler, F.S.; Buse, J.B.; Gu, Z. Microneedle-array patches loaded with hypoxia-sensitive vesicles provide fast glucose-responsive insulin delivery. *Proc Natl Acad Sci U S A* **2015**, *112*, 8260-8265, doi:10.1073/pnas.1505405112.
